# Current and future pangenomic research in cucurbit crops

**DOI:** 10.1270/jsbbs.24048

**Published:** 2025-02-26

**Authors:** Gentaro Shigita, Katsunori Tanaka, Kenji Kato

**Affiliations:** 1 Faculty of Life and Environmental Sciences, University of Tsukuba, 1-1-1 Tennodai, Tsukuba, Ibaraki 305-8572, Japan; 2 Department of Life Science Systems, TUM School of Life Sciences, Technical University of Munich, Emil-Ramann-Strasse 2, 85354 Freising, Germany; 3 Faculty of Agriculture and Life Science, Hirosaki University, 3 Bunkyo, Hirosaki, Aomori 036-8561, Japan; 4 Graduate School of Environmental, Life, Natural Science and Technology, Okayama University, 1-1-1 Tsushima-Naka, Kita-ku, Okayama 700-8530, Japan

**Keywords:** crop wild relatives, Cucurbitaceae, genetic resources, genome-wide association study, pangenome, presence/absence variation, structural variation

## Abstract

Pangenomics is the exploration and characterization of the full spectrum of genetic variation within a species or a given taxonomic clade. Driven by the accelerating decline in sequencing costs and the widespread adoption of long-read sequencing technologies, the “wave” of pangenomics is now hitting various major crops, uncovering substantial intraspecific diversity previously underestimated and neglected. This includes crops belonging to the gourd family (Cucurbitaceae), such as cucumber (*Cucumis sativus*), melon (*Cucumis melo*), watermelon (*Citrullus lanatus*), wax gourd (*Benincasa hispida*), and bottle gourd (*Lagenaria siceraria*), all of which are important on a global or regional scale. In this review, we consolidate the findings from all nine pangenomic studies reported as of June 2024, on the five cucurbit crops listed above. This summarizes the current state of pangenomics in the family. We then highlight remaining knowledge gaps for each crop, and propose further research to fill these gaps. Finally, we discuss how pangenomics will shape the future of crop breeding and expand the framework of crop genetic resources in synergy with other technological advances. These insights would apply not only to cucurbits but also to crops across diverse families.

## Introduction

A pangenome is defined as a non-redundant set of genes or genomic sequences present in a species or a given taxonomic clade, encompassing various types of genetic variations, such as single-nucleotide polymorphisms (SNPs) and structural variations (SVs), including insertions and deletions (indels), inversions, translocations, copy number variations (CNVs), and gene presence/absence variations (PAVs). Although substantial intraspecific diversity in plant genomes has been reported since the concept of pangenomes was first conceived in bacteria (Brunner *et al.* 2005, Scherrer *et al.* 2005, Tettelin *et al.* 2005, Yu *et al.* 2005), the application of pangenomics to plants has been hampered by the complex properties of their genomes, such as large size, abundance of repetitive sequences, and frequent polyploidization. Thanks to the exponential decrease in sequencing costs and recent advances in long-read sequencing technologies, sequencing and assembling multiple plant genomes is now feasible and affordable (Xie *et al.* 2024), marking a stark contrast to the first plant genome, which required immense effort, time, and financial investment (The Arabidopsis Genome Initiative 2000).

In the context of plant breeding, pangenomics holds great promise for linking diverse and complex phenotypic variations to their underlying genetic variations. An increasing number of studies across various crops have demonstrated the contribution of SVs to important agronomic traits, such as yield, nutritional content, flowering time, reproductive morphology, and responses to biotic and abiotic stresses (Yuan *et al.* 2021). Pangenomics is expected to accelerate the comprehensive identification of these genetic variations, leading to the rapid and efficient breeding of crop varieties with desirable traits. Encouraged by the democratization of plant genome sequencing described above, numerous pangenomic studies of major crops have been reported in recent years (e.g., Gao *et al.* 2019, He *et al.* 2023, Huang *et al.* 2023, Hufford *et al.* 2021, Jayakodi *et al.* 2020, Li *et al.* 2014, Montenegro *et al.* 2017, Tao *et al.* 2021, Walkowiak *et al.* 2020, Zhou *et al.* 2020). Moreover, several studies have constructed so-called “super-pangenomes” that include not only crop species but also their wild relatives, revealing untapped genetic diversity absent in the cultivated gene pool (e.g., Cochetel *et al.* 2023, Li *et al.* 2023b, Liu *et al.* 2023a, Shang *et al.* 2022, Stein *et al.* 2018, Wang *et al.* 2022, Zhang *et al.* 2021, Zhuang *et al.* 2022).

**Fig. 1.**
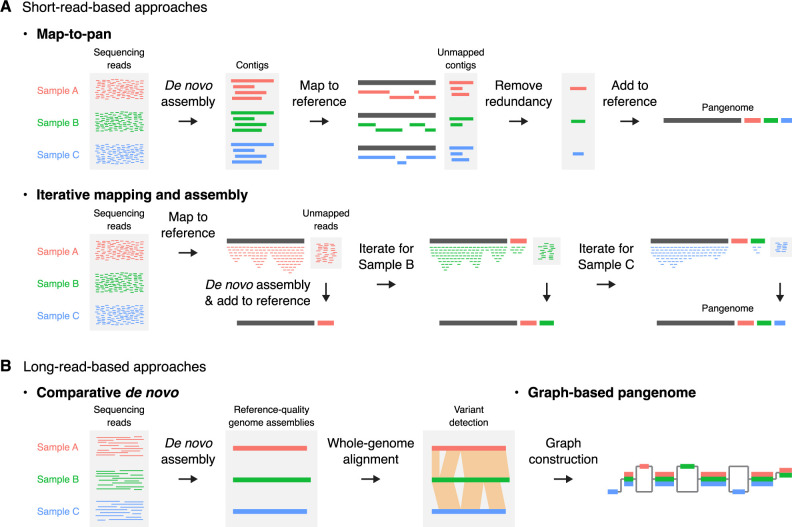
Schematic representation of typical short-read-based (A) and long-read-based (B) approaches used for pangenome construction.

Methodologically, there are several approaches to constructing a plant pangenome, each with its own advantages and disadvantages (Glick and Mayrose 2023, Golicz *et al.* 2016, Jiang *et al.* 2024). They can be categorized as short-read-based and long-read-based approaches, depending on the sequencing technology primarily relied upon (Fig. 1).

One commonly used short-read-based approach is the “map-to-pan” approach. In this approach, sequencing reads from each sample are first *de novo* assembled into contigs, which are then mapped to a reference genome. Unmapped contigs are considered non-reference sequences not present in the reference genome, clustered by sequence identity across all samples to remove redundancy, and then merged with the reference genome to construct the pangenome. Another commonly used short-read-based approach is the “iterative mapping and assembly” approach. Here, sequencing reads are first mapped to a reference genome, and then unmapped reads are *de novo* assembled into contigs that are added to the growing reference genome. This process is iterated for all samples to construct the pangenome. In both approaches, subsequent analysis typically involves remapping all sequencing reads to the constructed pangenome and identifying gene PAVs by calculating read coverage for each gene in each sample. While these short-read-based approaches offer scalability for large projects with hundreds or thousands of samples, the lack of precise information on the genomic coordinates of non-reference sequences generally limits their ability to detect SVs beyond gene PAVs and small indels.

Long-read-based approaches include the “comparative *de novo*” and “graph-based pangenome” approaches. Both construct reference-quality genome assemblies for all samples, using long-read sequencing technology from Pacific Biosciences (PacBio) and/or Oxford Nanopore Technologies (ONT). The comparative *de novo* approach aligns and compares these genome assemblies to identify genetic variations. The graph-based pangenome approach goes even further, by integrating the sequences and coordinates of the identified SVs into a graph structure. This structure includes both reference and alternative allele sequences, enabling proper mapping of short reads derived from regions adjacent to SVs and accurate genotyping of all types of genetic variation at the population scale. However, the requirement for extensive long-read data and expensive computational resources limits the application of these long-read-based approaches to relatively small-scale studies, typically involving up to 100 samples with small genome sizes.

The gourd family (Cucurbitaceae), commonly referred to as cucurbits, comprises 95 genera with about 1,000 species (Christenhusz and Byng 2016, Renner and Schaefer 2016, Schaefer and Renner 2011). Numerous cucurbits have economic value as global, regional, or local agricultural crops (Chomicki *et al.* 2020). The most economically important crops in the family include cucumber (*Cucumis sativus*), melon (*Cucumis melo*), watermelon (*Citrullus lanatus*), and various types of zucchini, squash, and pumpkin (*Cucurbita pepo*, *maxima*, and *moschata*). All are grown worldwide, primarily for their mature or immature fruits or in some cases for their young leaves, seeds, and oils. Regionally important crops include wax gourd (*Benincasa hispida*), bottle gourd (*Lagenaria siceraria*), sponge gourd (*Luffa aegyptiaca*), ridge gourd (*Luffa acutangula*), bitter gourd (*Momordica charantia*), chayote (*Sechium edule*), and snake gourd (*Trichosanthes cucumerina*). Minor crops grown in local communities include tinda (*Benincasa fistulosa*), preserving melon (*Citrullus amarus*), colocynth (*Citrullus colocynthis*), egusi melon (*Citrullus mucosospermus*), ivy gourd (*Coccinia grandis*), anchote (*Coccinia abyssinica*), West Indian gherkin (*Cucumis anguria*), kiwano (*Cucumis metuliferus*), caigua (*Cyclanthera pedata*), cassabanana (*Sicana odorifera*), monk fruit (*Siraitia grosvenorii*), fluted pumpkin (*Telfairia occidentalis*), etc.

As with major crops in other families, pangenomic studies of cucurbit crops have progressed rapidly over the past few years. In this review, we first describe the current state of pangenomic research in cucurbit crops by consolidating key findings from nine studies. Next, we highlight knowledge gaps that remain for each crop, and propose further research to fill these gaps. Finally, we discuss the future directions of plant pangenomic research and its implications for the framework of crop genetic resources, which would apply not only to cucurbits but also to crops from other families.

## Cucurbit pangenomes today

This section lists all nine pangenomic studies of five cucurbit crops reported as of June 2024 (Table 1) and outlines their sampling, methodologies, and key findings to provide an overview of the current state of pangenomic research in the gourd family.

### Cucumber

Cucumber, domesticated in the Himalayan foothills approximately 3,000 years ago, has evolved into one of the most economically important vegetable crops, cultivated and consumed worldwide (Chomicki *et al.* 2020, Renner *et al.* 2007, Sebastian *et al.* 2010). Its prominence in agriculture is highlighted by its distinction as the ninth plant and the first cucurbit to undergo genome sequencing (Huang *et al.* 2009, Michael and Jackson 2013). Continuing this trend, cucumber also paved the way for the realm of pangenomics in cucurbit crops.

**Fig. 2.**
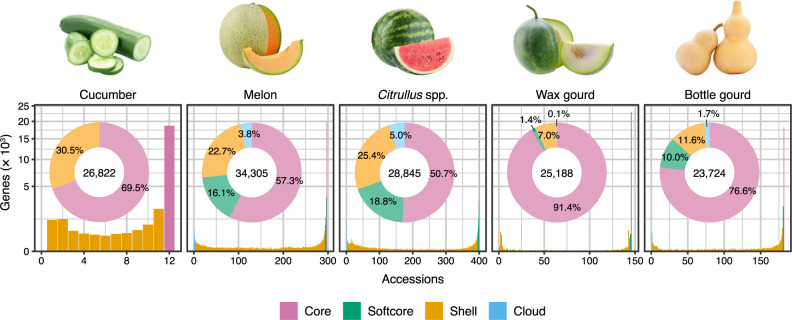
Pangenomic landscape of cucurbit crops. Histograms show how many genes are shared among different numbers of accessions of cucumber (Li *et al.* 2022), melon (Sun *et al.* 2022), *Citrullus* spp. (Sun *et al.* 2023), wax gourd (Yang *et al.* 2023), and bottle gourd (Zhao *et al.* 2024). Genes are classified into four categories according to their presence frequency: core (shared by all accessions), softcore (shared by >99% of accessions), shell (shared by 1–99% of accessions), and cloud (present in <1% of accessions). Donut charts illustrate the proportion of genes in each category, with the total number of genes in the pangenome shown in the center.

The first and only pangenomic study of cucumber reported to date that considers the entire nuclear genome is centered on the genome assemblies of 12 accessions (Table 1). Using a combination of PacBio long reads and Illumina short reads, Li *et al.* (2022) newly constructed chromosome-scale assemblies for 11 representative accessions selected from 115 accessions according to their phylogenetic relationships (Lv *et al.* 2012, Qi *et al.* 2013). These 11 accessions represent all four genetic and geographic groups of cucumber (East Asia, Eurasia, Xishuangbanna, India) identified in previous studies (Lv *et al.* 2012, Qi *et al.* 2013), and include three accessions of wild cucumber (*C. s.* var. *hardwickii*), from which cultivated cucumber (*C. s.* var. *sativus*) was domesticated (Bisht *et al.* 2004, Qi *et al.* 2013). With the addition of the reference genome of the ‘Chinese long’ inbred line 9930 (Li *et al.* 2019), 12 chromosome-scale genome assemblies were meticulously aligned and compared using nucmer and delta-filter in the MUMmer software (Marçais *et al.* 2018), axtChain and chainNet (Kent *et al.* 2003), and diffseq from the EMBOSS package (Rice *et al.* 2000). This analysis identified over 4.3 million genetic variations, including 56,214 SVs. Additionally, seven megabase-scale inversions were identified between the wild accessions and the cultivated accessions, six of which had been reported in a previous study based on fluorescence *in situ* hybridization (Yang *et al.* 2012). A gene PAV-based pangenome was constructed by clustering a total of 299,692 gene models predicted in the 12 genomes using GET_HOMOLOGUES-EST (Contreras-Moreira *et al.* 2017). The resulting 26,822 gene clusters consisted of 18,651 (69.5%) core clusters present in all 12 accessions, and 8,171 (30.5%) variable clusters absent in at least one accession (Fig. 2). Genes in the core clusters tended to have longer gene lengths, higher expression levels, and lower non-synonymous/synonymous substitution ratios (i.e., slower evolutionary rates), compared to genes in the variable clusters.

**Fig. 3.**
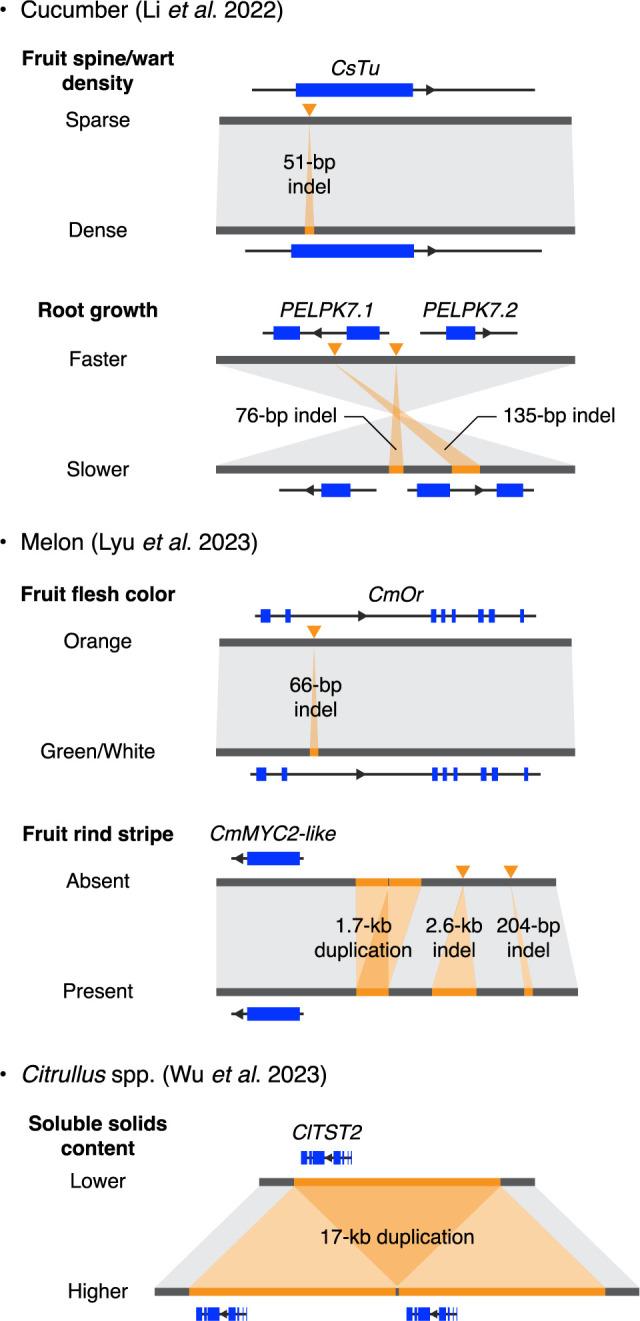
Representative examples of structural variations associated with agronomic traits, identified through pangenomic research in cucurbit crops.

To enable accurate genotyping of SVs at the population scale, a graph-based pangenome was constructed using the vg toolkit (Garrison *et al.* 2018) by integrating sequences and coordinates of the identified SVs into the reference genome of 9930. The SVs among 115 accessions were genotyped by mapping their short-read resequencing data (Qi *et al.* 2013) to this graph-based pangenome and subsequently used for SV-based genome-wide association study (GWAS) and screening to identify genes associated with agronomic traits, such as female flower rate on a primary branch, fruit spine/wart density, branch number, flowering time, and root growth. Besides rediscoveries of previously reported genes responsible for these traits, two genes were newly identified as likely responsible for faster root growth of cultivated cucumbers, compared to wild cucumbers (Fig. 3). Both genes are homologs of *Arabidopsis PELPK1*, which encodes a positive regulator of root development (Rashid and Deyholos 2011), and are therefore designated as *PELPK7.1* (*Csa9930_7G006910*) and *PELPK7.2* (*Csa9930_7G006920*). Primary root lengths and weights were generally in accordance with the expression levels of *PELPK7.1* and *PELPK7.2*, with higher expression levels linked to longer primary root lengths and higher root weights. Furthermore, a clear reduction of nucleotide diversity (π) in cultivated accessions was observed around these genes, suggesting that this region underwent strong selection during the domestication of cucumber. All these findings demonstrate the usefulness of the graph-based pangenome in identifying genetic variations associated with agronomic traits in cucumber biology and genetic research.

The same set of 12 cucumber genomes was used in studies of specific gene families, such as *WOX* (Yin *et al.* 2023), *TIFY* (Liu *et al.* 2023b), and *ZIP* (Wang *et al.* 2024), to investigate their conservation and divergence among different cucumber accessions. Also, a pangenomic study focusing exclusively on the chloroplast genome has been reported, in which the complete chloroplast genomes of 50 cucumber accessions were assembled and compared, leading to the identification of chloroplast genes differentially expressed under high- and low-temperature stresses (Xia *et al.* 2023).

### Melon

Melon is another important horticultural crop cultivated worldwide in the genus *Cucumis*. During its cultivation history of at least 4,000 years, different varieties have been selected in each region, resulting in a spectacular diversity of fruit traits (Kirkbride 1993, Pitrat 2016, Pitrat *et al.* 2000). The origin of melon domestication had long been considered to be Africa, due to the abundance of wild *Cucumis* species (Robinson and Decker-Walters 1997, Whitaker and Davis 1962). However, phylogenetic studies based on genetic polymorphisms in the chloroplast and nuclear genomes have consistently indicated that melons were domesticated multiple times, independently, in Africa and South Asia (Endl *et al.* 2018, Liu *et al.* 2020, Shigita *et al.* 2023, Tanaka *et al.* 2013, 2025, Wang *et al.* 2021, Zhao *et al.* 2019). These multiple origins of melon make its pangenome of particular interest among cucurbit crops, and four pangenomic studies have been reported to date (Table 1).

The first published pangenomic study of melon, by Sun *et al.* (2022), is based on a reanalysis of short-read resequencing data of 297 accessions generated in an earlier study (Liu *et al.* 2020). The melon pangenome was constructed by the map-to-pan approach using tools such as MEGAHIT (Li *et al.* 2015), CD-HIT (Fu *et al.* 2012), MUMmer (Marçais *et al.* 2018), and BLAST (Camacho *et al.* 2009), with the genome sequence of the doubled-haploid line DHL92 (Garcia-Mas *et al.* 2012) as a reference, resulting in the identification of 168 Mb non-reference sequences harboring 4,325 protein-coding genes. The gene PAVs among the 297 accessions were identified by mapping reads to the pangenome using BWA (Li and Durbin 2009) and calculating the read coverage of each gene in each accession using ccov from the HUPAN pipeline (Duan *et al.* 2019). Of 34,305 protein-coding genes in the pangenome, 19,660 (57.3%) were identified to be core genes and 14,645 were identified to be variable genes (Fig. 2). The variable genes were further classified into 5,540 softcore genes, 7,788 shell genes, and 1,317 cloud genes shared among more than 99%, 1–99%, and less than 1% of the accessions, respectively. Comparison of gene presence frequencies between landraces and improved varieties showed that more genes were lost than were gained during improvement in both subspecies of melon, subsp. *melo* and subsp. *agrestis*. A gene PAV-based GWAS was performed based on the shell genes. It identified 13 gene PAVs associated with length, shape, and width of fruit, four of which were non-reference genes not represented in the reference genome of DHL92. The melon pangenome was also used in a comprehensive search for resistance gene analogs (RGAs), resulting in the identification of 709 RGAs. Of these, 106 were identified as variable genes, potentially explaining differences in disease resistance among melon accessions.

The second pangenomic study of melon, by Oren *et al.* (2022), centered on *de novo* assembly of the genomes of 25 founder accessions, using a combination of ONT long reads and Illumina short reads. These 25 founder accessions consisted of 17 accessions of subsp. *melo*, seven accessions of subsp. *agrestis*, and one feral accession collected in central Israel. They were selected by integrative consideration of taxonomic classification and phenotypic and genotypic variations from 177 inbred lines used in an earlier study (Gur *et al.* 2017). Genome assemblies of the 25 founder accessions were constructed through *de novo* assembly of ONT long reads into contigs, polishing with Illumina short reads, and scaffolding guided by the reference genome of a Japanese cultivar ‘Harukei-3’ (Yano *et al.* 2020). The assembly sizes ranged from 358 Mb to 374 Mb, with significantly smaller sizes in subsp. *agrestis* accessions than in subsp. *melo* accessions. Alignment and comparison of the 25 genomes using MUMmer (Marçais *et al.* 2018) and Assemblytics (Nattestad and Schatz 2016) revealed extensive genomic variation, with approximately 300,000 SVs and nine million SNPs identified. To fully exploit this genetic diversity for agronomic trait dissection, 300 distinct F_2_ populations were developed by self-pollinating F_1_ individuals derived from half-diallel crosses of the 25 founder accessions (Dafna *et al.* 2021). The implementation of this unified framework was demonstrated through genetic dissections for various traits, including mottled rind, rind color intensity, fruit sugar content, and resistance to *Fusarium* wilt and *Fusarium* root and stem rot.

The third pangenomic study of melon resulted in the construction of a graph-based pangenome (Vaughn *et al.* 2022). Using the PacBio long-read sequencing technology, genome assemblies were newly constructed for two parental accessions: MR-1, a multi-disease resistant breeding line derived from an Indian landrace PI 124111 (Thomas 1986), and Ananas Yoqne’am, an Israeli heirloom cultivar (Paris *et al.* 2013). Together with the reference genome of DHL92, multiple sequence alignments of three genomes were generated for each chromosome using progressiveMauve (Darling *et al.* 2010), enabling the construction of a graph-based pangenome using the vg toolkit (Garrison *et al.* 2018). Their workflow is publicly available as PanPipes (https://github.com/USDA-ARS-GBRU/PanPipes). The power of this graph-based pangenome was assessed through genotyping of 149 recombinant inbred lines derived from the two parents, using their skim-seq (i.e., low-depth whole genome sequencing) reads and previously generated genotyping-by-sequencing reads (Branham *et al.* 2018). The results demonstrated that the graph-based pangenome can aid in genotyping 19% more variants, with an error rate two or three times lower, compared to the conventional approach of relying on a single reference genome. The utility of the graph-based pangenome was further demonstrated through variant profiling of genes responsible for resistance to *Fusarium* wilt (races 1 and 2) and powdery mildew. In many of these cases, association analysis and comparative genomic analysis implied that functional mutation is likely involved in large SVs that have been difficult to detect when using short reads and a single reference genome.

The fourth pangenomic study of melon, by Lyu *et al.* (2023), assembled the genome of the Chinese landrace ‘Mapao’ at the chromosome scale, using a combination of PacBio long-read sequencing and high-throughput chromosome conformation capture (Hi-C) technologies and comparing to eight previously reported melon genomes (Ling *et al.* 2021, Oren *et al.* 2022, Pichot *et al.* 2022, Ruggieri *et al.* 2018, Yang *et al.* 2020, Yano *et al.* 2020, Zhang *et al.* 2019). Clustering all gene models predicted in the nine genomes using OrthoFinder (Emms and Kelly 2019) resulted in 36,617 gene clusters, comprising 12,290 (33.6%) core clusters containing all nine accessions and 24,327 (66.4%) variable clusters missing at least one accession. Higher non-synonymous/synonymous substitution ratios (i.e., faster evolutionary rates) were shown for variable genes compared to core genes, suggesting a potential role of these variable genes in the diverse fruit morphologies of melon. Sequence comparisons using MUMmer (Marçais *et al.* 2018) and SyRI (Goel *et al.* 2019) uncovered over three million SVs among the nine genomes. This information was embedded into a graph-based pangenome using the vg toolkit (Garrison *et al.* 2018), which was used for the genetic dissection of various fruit traits, including sucrose content, flesh color and thickness, rind stripe and suture, and exocarp edibility, based on several bi-parental populations (Fig. 3). Notably, *CmPIRL6*, a gene predicted to encode a leucine-rich repeat protein, was newly identified as a candidate gene responsible for the edibility of melon fruit exocarp.

### Watermelon

Watermelon is cultivated in temperate and tropical regions of the world, and is a source of water and nutrients for animals and humans (Paris 2015, Wehner 2008). Archaeological evidence indicates that watermelon was domesticated in northeastern Africa over 4,000 years ago and was most likely spread by African desert nomads. This hypothesis was recently strengthened by the genomic analysis of a Sudanese landrace with a non-bitter and whitish pulp, known as Kordofan melon (*C. lanatus* subsp. *cordophanus*), which was found to be the closest relative and a possible progenitor of domesticated watermelons (Renner *et al.* 2021). Besides *C. lanatus*, the genus *Citrullus* includes six other species (Chomicki and Renner 2015), some of which are cultivated from Africa to the Mediterranean region for their fruits, seeds, forage, medicinal properties, and oil (Chomicki *et al.* 2020). Moreover, *C. amarus* and *C. mucosospermus* are readily cross-compatible with watermelon and have been used in watermelon breeding as sources of disease and pest resistance (Levi *et al.* 2017). Reflecting the agricultural values of these close relatives of watermelon, the two pangenomic studies of watermelon reported so far are both so-called super-pangenomic studies that include not only *C. lanatus* but also other *Citrullus* species (Table 1).

The first pangenome of watermelon, constructed by Sun *et al.* (2023), is based on a reanalysis of previously generated short-read resequencing data from 400 *Citrullus* accessions (Guo *et al.* 2019) (Table 1). These 400 accessions represent all seven extant species in the genus, consisting of 335 of *C. lanatus*, 28 of *C. amarus*, 18 of *C. mucosospermus*, 15 of *C. colocynthis*, two of *C. rehmii*, and one each of *C. ecirrhosus* and *C. naudinianus*. Following the map-to-pan approach, the short reads of each accession were *de novo* assembled separately into contigs using MEGAHIT (Li *et al.* 2015), and then mapped to the reference genome of a Chinese inbred line ‘97103’ (Guo *et al.* 2019) using MUMmer (Marçais *et al.* 2018). After removing redundancy and contamination, a total of 477 Mb of non-reference sequences containing 6,249 protein-coding genes were identified and merged with the reference genome to construct a super-pangenome. Despite the much higher number of accessions for *C. lanatus*, only 64 Mb of the non-reference sequences were from *C. lanatus*, while the remaining 413 Mb were from the other six species. Gene PAVs among the 400 accessions were called by mapping all reads to the super-pangenome using Bowtie 2 (Langmead and Salzberg 2012) and calculating the read coverage per gene per accession using SGSGeneLoss (Golicz *et al.* 2015). Of the 28,845 protein-coding genes in the super-pangenome, 14,613 were classified as core genes (shared by all accessions), 5,437 as softcore genes (present in over 99% of accessions), 7,341 as shell genes (present in 1–99% of accessions), and 1,454 as cloud genes (present in less than 1% of accessions) (Fig. 2). Favorable and unfavorable genes were identified by comparing gene PAVs between *C. lanatus* and its sister species, *C. mucosospermus*, indicating that many genes were disproportionally lost during the domestication and improvement of watermelon. A total of 661 RGAs were identified in the super-pangenome, including many variable non-reference genes. Some RGAs were found to be located within three known quantitative trait loci (QTL) intervals for resistance to gummy stem blight (Gimode *et al.* 2021). A gene PAV-based GWAS was performed based on the shell genes, and identified eight gene PAVs associated with fruit flesh color, which were not detected in the SNP-based GWAS performed in an earlier study (Guo *et al.* 2019). Furthermore, a comparison of gene PAVs between populations with different fruit flesh colors revealed four non-reference genes predicted to be associated with carotenoid accumulation. These genes were present at significantly higher frequencies in the white flesh population and appeared to negatively regulate carotenoid accumulation in the fruit flesh.

Another pangenomic study of watermelon paid more attention to the wild side of the genus, where reference genomes were newly assembled for *C. mucosospermus*, *C. amarus*, and *C. colocynthis*, using a combination of PacBio long reads and Illumina short reads or solely Illumina short reads (Wu *et al.* 2023). Comparison of these genomes with three previously assembled genomes of *C. lanatus* (Guo *et al.* 2019, Renner *et al.* 2021, Wu *et al.* 2019) revealed a large inter-chromosomal rearrangement involving chromosomes 1 and 4 between *C. colocynthis* and the other three species, *C. lanatus*, *C. amarus*, and *C. mucosospermus*. Overall collinearity was found between chromosome 4 of *C. colocynthis* and chromosome 8 of melon, suggesting that *C. colocynthis* preserves ancestral karyotype and that these rearrangements occurred after the divergence of *C. colocynthis* from other species and before the speciation of *C. amarus* from a common ancestor of *C. mucosospermus* and *C. lanatus*. A *Citrullus* genus super-pangenome was constructed using resequencing data from 547 accessions, including 201 newly sequenced wild accessions and 346 from an earlier study (Guo *et al.* 2019). These 547 accessions comprise 349 *C. lanatus*, 31 *C. mucosospermus*, 131 *C. amarus*, and 36 *C. colocynthis*. Species-level pangenomes were first constructed through the map-to-pan approach using tools such as SPAdes (Prjibelski *et al.* 2020), QUAST (Gurevich *et al.* 2013), CD-HIT (Fu *et al.* 2012), and BLAST (Camacho *et al.* 2009), with the respective reference genome for each species. A total of 24.5 Mb, 15.6 Mb, 18.3 Mb, and 42.4 Mb of non-reference sequences were identified in *C. lanatus*, *C. mucosospermus*, *C. amarus*, and *C. colocynthis*, respectively, harboring 2,288, 583, 1,922, and 2,521 non-reference genes that are absent in the respective reference genomes. Gene PAVs were called by mapping the resequencing reads of each accession to the pangenome of its own species using BWA (Li and Durbin 2009) and calculating the read coverage of each gene using BEDTools (Quinlan and Hall 2010). These four species-level pangenomes were subsequently combined into a super-pangenome based on syntenic relationships among the species determined using Liftoff (Shumate and Salzberg 2021), BLAT (Kent 2002), and MCScanX (Wang *et al.* 2012). Of the 37,957 genes in the *Citrullus* super-pangenome, 24,235 (63.7%) were core genes present in all accessions. The proportion of core genes was much lower in the super-pangenome than in the species-level pangenomes (85.6–97.2%), highlighting diverse gene repertoires among the four species. By comparing the genomes of cultivated watermelon and Kordofan melon (*C. lanatus* subsp. *cordophanus*), recently identified as the possible direct progenitor of cultivated watermelon (Renner *et al.* 2021), a tandem duplication was identified of the *Tonoplast Sugar Transporter 2* (*ClTST2*) gene (Fig. 3), which is known to regulate sugar accumulation in watermelon (Ren *et al.* 2018). This *ClTST2* tandem duplication was predominant in watermelon landraces and almost fixed in watermelon cultivars. The soluble solid contents of fruit flesh were significantly higher in accessions carrying the *ClTST2* tandem duplication than in those with only one copy. These results suggest that the *ClTST2* tandem duplication was selected during the domestication and improvement of watermelon, likely due to its important role in promoting sugar accumulation in fruits.

### Wax gourd

The wax gourd is an important vegetable crop, particularly in Asian countries, owing to its high yield, ease of cultivation, extremely long shelf-life, and several medicinal properties (Dhillon *et al.* 2017, Marr *et al.* 2007). Archaeological evidence from Thailand suggests its cultivation history dates back as far as 10,000 years ago (Chomicki *et al.* 2020, Pyramarn 1989), with the Indo-China region being regarded as its center of origin (Robinson and Decker-Walters 1997).

The pangenomic study of wax gourd by Yang *et al.* (2023) is based on a reanalysis of the short-read resequencing data from 146 accessions (Xie *et al.* 2019) (Table 1). Through the map-to-pan approach using tools such as MaSuRCA (Zimin *et al.* 2013), MUMmer (Marçais *et al.* 2018), CD-HIT (Fu *et al.* 2012), and BLAST (Camacho *et al.* 2009), the authors identified 136 Mb of novel sequences containing 996 genes not present in the reference genome of an inbred line B227 (Xie *et al.* 2019). These novel sequences were merged with the reference genome to construct the wax gourd pangenome, and gene PAVs were called using BWA (Li and Durbin 2009) and SGSGeneLoss (Golicz *et al.* 2015). Of the 25,188 genes in the pangenome, 23,034 (91.4%) were core genes present in all 146 accessions, and 2,154 (8.6%) were variable genes absent in at least one accession (Fig. 2). Comparisons of gene PAV frequency among different wax gourd populations with varying degrees of domestication revealed that over 300 genes, including many presumably involved in defense responses, were lost during the domestication and improvement of wax gourd. Using the gene PAV information from 779 shell genes present in 1–99% of accessions, a gene PAV-based GWAS was performed for four traits related to fruit size (length, diameter, weight, and flesh thickness), and identified five gene PAVs significantly associated with these traits. Additionally, the wax gourd pangenome was used to comprehensively identify specific gene families, including RGAs and transcription factors such as *NAC*, *WRKY*, and *HSF*, highlighting their conservation and variability among different wax gourd accessions.

### Bottle gourd

Bottle gourd is native to Africa and is one of the earliest domesticated crops, with a cultivation history of over 11,000 years (Chomicki *et al.* 2020, Erickson *et al.* 2005, Richardson 1972). The long and close relationship between humans and bottle gourd is reflected in its diverse uses: wild-type varieties with bitter pulp are used for medicines, ornaments, containers, and musical instruments, while non-bitter varieties are used for their pulp and young shoots as vegetables (Dhillon *et al.* 2017). It is also used as a rootstock for other cucurbit crops, such as watermelon, to impart disease and stress tolerance or to avoid replant failure (Davis *et al.* 2008, Mashilo *et al.* 2017). Archaeological evidence indicates the widespread distribution of bottle gourd in both Eurasia and the Americas since pre-Columbian times (Clarke *et al.* 2006, Richardson 1972, Schlumbaum and Vandorpe 2012). A phylogenetic study of both archaeological and living specimens proposed that the species was brought from Africa to Eurasia with human migration, but reached the Americas via transoceanic drift across the Atlantic from Africa (Kistler *et al.* 2014).

To elucidate the genetic diversity, demographic history, and the genetic basis of local adaptations of bottle gourd, Zhao *et al.* (2024) performed genomic and pangenomic analyses using short-read resequencing data from 197 accessions, consisting of 146 newly sequenced worldwide representative accessions and 51 landraces sequenced in an earlier study (Xu *et al.* 2021) (Table 1). Through conventional genomic analysis, which maps sequencing reads from each accession to the reference genome of an inbred line USVL1VR-Ls (Wu *et al.* 2017), 1,681,089 SNPs and 643,569 small indels were identified. Phylogenetic and population structure analyses based on 17,967 biallelic SNPs at four-fold degenerate sites divided the bottle gourd accessions into five populations, highly consistent with their geographic distributions in Africa, America, Around Aegean, South Asia, and East Asia.

Analyses based on population genetic indices and demographic modeling reconstructed the domestication and dispersal history of bottle gourd, indicating it was domesticated in sub-Saharan Africa around 12,500 years ago. Domesticated bottle gourd was then spread to the New World via Atlantic drift and to Eurasia with human migration in the early Holocene, aligning well with scenarios proposed in earlier studies based on archaeological remains (Erickson *et al.* 2005, Kistler *et al.* 2014). The high-resolution SNP data were also used for a GWAS for zucchini yellow mosaic virus (ZYMV) resistance, leading to the identification of three distinct association signals on chromosomes 1, 7, and 9, with candidate genes reported to be involved in plant immunity in other species.

To unveil the entire gene repertoire within the species that cannot be accessed by conventional genomic analysis, the bottle gourd pangenome was constructed through the map-to-pan approach using MEGAHIT (Li *et al.* 2015), QUAST (Gurevich *et al.* 2013), CD-HIT (Fu *et al.* 2012), and BLAST (Camacho *et al.* 2009). This identified 59.7 Mb sequences harboring 1,534 protein-coding genes absent from the reference genome of USVL1VR-Ls. Gene PAVs were called using BWA (Li and Durbin 2009) and SGSGeneLoss (Golicz *et al.* 2015). Of the 23,724 genes in the bottle gourd pangenome, 18,184 (76.6%) were core genes shared among all accessions, while 5,540 (23.4%) were variable genes (Fig. 2). The most ancestral African population harbored a higher number of genes, particularly those absent in the reference genome, compared to the other four populations, indicating that a considerable number of genes were lost during the global dispersal and subsequent improvements of bottle gourd. Analysis of the gene PAV atlas identified 503 genes present at significantly different frequencies among the populations. Functional predictions of these genes revealed a substantial enrichment of defense-related genes, such as the *Mildew Locus O* (*MLO*) family, emphasizing their pivotal role in adaptations to differing environmental conditions (Kusch and Panstruga 2017).

## Remaining gaps

Based on the current state of cucurbit pangenomes outlined in the previous section, this section highlights the remaining knowledge gaps for each crop and proposes further genomic or pangenomic studies to address those gaps. It also mentions other cucurbit crops for which no pangenomic studies have been reported so far.

### Cucumber

A quite narrow genetic diversity of cucumber had been claimed by classical studies based on dozens of isozyme loci or random amplified polymorphic DNA (RAPD) markers (Horejsi and Staub 1999, Meglic and Staub 1996, Meglic *et al.* 1996, Staub *et al.* 1997, 1999). Still, genome-wide studies enabled by short-read sequencing technology have revealed millions of SNPs, hundreds of thousands of small indels, and tens of thousands of SVs among 115 worldwide cucumber accessions (Qi *et al.* 2013, Zhang *et al.* 2015). This raises the question of whether the 12 accessions used by Li *et al.* (2022), although selected in consideration of phylogenetic relationships, are sufficiently representative of the intraspecific variation of cucumber. Several core collections—diversity panels systematically selected to cover most of the genetic diversity—have been developed from different sets of cucumber genetic resources (Lv *et al.* 2012, Shigita *et al.* 2024, Staub *et al.* 2002, Wang *et al.* 2018). These core collections would be appropriate subjects for research aimed at a more comprehensive understanding of the cucumber pangenome.

Despite its pioneering role in genomic research within the Cucurbitaceae family, the genome size of cucumber remains a mystery. The assembly sizes of the two most commonly used reference genomes, 9930 (Li *et al.* 2019) and Gy14 (Yang *et al.* 2012), along with the 11 genomes assembled by Li *et al.* (2022), are all around 250 Mb. However, estimates based on flow cytometry and *k*-mer frequencies suggest much larger genome sizes of approximately 350 Mb (Arumuganathan and Earle 1991, Huang *et al.* 2009). This large discrepancy implies that up to 100 Mb of sequences are missing in many assembled genomes, or that there is substantial intraspecific variation in the genome size of cucumber. Recently, several genome assemblies have been constructed with sizes closer to these estimates, revealing an abundance of long tandem repeats in the cucumber genome (Guan *et al.* 2024, Osipowski *et al.* 2020, Seiko *et al.* 2025, Turek *et al.* 2023). Given that state-of-the-art sequencing, assembly, and scaffolding technologies have enabled genome assemblies at the telomere-to-telomere scale in other cucurbits, such as melon (Li *et al.* 2023a, Mo *et al.* 2024, Wei *et al.* 2023), watermelon (Deng *et al.* 2022), and bitter gourd (Fu *et al.* 2022), constructing such an assembly in cucumber could help to solve the mystery of its genome size.

Looking beyond the species, a cross-species study including the sister species of cucumber, *C. hystrix*, would be of great interest. *C. hystrix* grows wild in the Himalayan foothills, where cucumber originated, and is the only species that shows reproducible cross-compatibility with cucumber (Joseph John *et al.* 2018). A synthetic allotetraploid, *C.* × *hytivus*, has been produced between cucumber and *C. hystrix* through interspecific hybridization via embryo rescue and subsequent chromosome doubling (Chen and Kirkbride 2000, Chen *et al.* 1997). Several introgression lines were developed through recurrent backcrossing of this synthetic allotetraploid to cucumber (Zhou *et al.* 2009), with some exhibiting substantial improvements in disease resistance (Delannay *et al.* 2010). This demonstrates the unique and practical value of *C. hystrix* in expanding the genetic diversity of cucumber through conventional crossbreeding. Although chromosome-scale reference genomes are available for both *C. hystrix* (Qin *et al.* 2021) and *C.* × *hytivus* (Yu *et al.* 2021), the genetic diversity within *C. hystrix* has not yet been assessed and compared to that of cucumber. Comparisons between the two species have also been of interest regarding karyotype dynamics. Among *Cucumis* species with known chromosome numbers, cucumber is the only one where 2*n* = 14, while other species, including *C. hystrix*, have 2*n* = 24 or a multiple thereof (Kirkbride 1993). Although comparative genomic and cytogenetic studies relying on a few individuals of cucumber and *C. hystrix* have modeled the chromosomal rearrangement events underlying the descending dysploidy of cucumber (Yang *et al.* 2014, Zhao *et al.* 2021), a study at the population scale may provide a more detailed picture of these drastic chromosomal changes. For these reasons, a super-pangenomic study involving multiple accessions of cucumber and *C. hystrix* would be highly valuable from both applied and fundamental perspectives.

### Melon

All four pangenomic studies of melon outlined above focused exclusively on two of the three major lineages within the species, both of which are thought to have been domesticated on the Indian subcontinent, and did not include a single accession from the third lineage endemic to the African continent (Shigita *et al.* 2023, Zhao *et al.* 2019). This third lineage includes the landraces known as ‘Tibish’ and ‘Fadasi’ grown in the Sudanese region and officially described as subsp. *meloides* (Endl *et al.* 2018, Mohamed and Pitrat 1999). There is not even a reference genome available for this subspecies, likely due to its marginal market value worldwide. However, several studies have shown the non-negligible genetic diversity of African accessions, suggesting their immense value as breeding material (Tanaka *et al.* 2013, 2025, Wang *et al.* 2021). Therefore, further studies including subsp. *meloides* are essential for a comprehensive understanding of intraspecific genomic diversity in melon. For this purpose, the core collections developed in previous studies would be suitable resources because they have been systematically selected to represent intraspecific genetic diversity and include several African accessions that are most likely to be subsp. *meloides* (Shigita *et al.* 2023, Wang *et al.* 2021).

In contrast to cucumber and watermelon, where cross-compatible wild species have been found, no reproducible interspecific hybridization has been reported in melon (Chen and Adelberg 2000), leaving the direct progenitor of cultivated melon unclear. The two latest phylogenetic studies of the genus *Cucumis* have identified *C. picrocarpus* from Australia and *C. trigonus* from India as the closest relatives of *C. melo* (Endl *et al.* 2018, Sebastian *et al.* 2010). A super-pangenomic study of the genus *Cucumis*, including these two closest relatives along with other representative wild species, may provide new insights into the history of melon domestications.

### Watermelon

In terms of sampling, the two pangenomic studies of watermelon reported to date are considered the most extensive among cucurbits, with a larger number of accessions than for other cucurbit crops and including wild relatives of the same genus (Table 1). These extensive samplings may have contributed to the highest proportion of variable genes among the five cucurbit crops (Fig. 2). On the other hand, although Wu *et al.* (2023) used PacBio long reads to assemble the reference genome of *C. mucosospermus*, the pangenome construction itself relied on the map-to-pan approach using only short reads in both pangenomic studies of watermelon, and thus limiting their representativeness of SVs to gene PAVs only. This leaves an opportunity for more comprehensive studies in terms of methodology, such as those conducted in cucumber and melon using long-read sequencing technologies.

Further research opportunities arise from the need for increased sampling and enhanced genomic resources for wild relatives of watermelon. Although both watermelon pangenomes constructed to date are super-pangenomes encompassing multiple *Citrullus* species, their samplings are skewed toward *C. lanatus*. *C. ecirrhosus*, *C. naudinianus*, and *C. rehmii* are particularly underrepresented, as only four accessions of these species were used by Sun *et al.* (2023). Recently, a chromosome-scale genome of *C. naudinianus* was assembled (Jarret 2021), leaving only *C. ecirrhosus* and *C. rehmii* without a genomic resource in the genus. A comparative genomic study of all seven extant species of *Citrullus* would deepen our understanding of the evolutionary history of this important genus and elucidate the genetic mechanisms underlying specific traits unique to certain species, such as the absence of tendrils in *C. ecirrhosus* and dioecy in *C. naudinianus*.

### Wax gourd

While the pangenome constructed by Yang *et al.* (2023) revealed a repertoire of gene PAVs among 146 accessions of wax gourd, their short-read-based analyses did not cover other types of SVs, as in the case of watermelon. The genome size of wax gourd is about 900 Mb (Bennett *et al.* 1982), more than twice the genome size of most cucurbit crops, which are typically smaller than 400 Mb. The absence of traces of whole-genome duplication in the wax gourd genome indicates that this exceptionally large genome size is not due to polyploidization but rather to the explosive amplification of transposable elements, which account for nearly three-quarters of the genome (Luo *et al.* 2023, Xie *et al.* 2019). A study using long-read sequencing technologies would enable us to genotype PAVs of individual transposon copies, typically seen as indels of a few kilobases, and provide a more comprehensive view of the wax gourd pangenome.

In addition, looking outside the species, there are several opportunities for further exploration. The only other accepted species in the genus *Benincasa*, *B. fistulosa* (commonly known as tinda), is cultivated in India and Pakistan for its immature fruits as a vegetable (Chomicki *et al.* 2020, Renner and Pandey 2013, Schaefer and Renner 2011). Although the cross-compatibility between this species and wax gourd has not been examined, *B. fistulosa* is a potential resource to broaden the gene pool of wax gourd. A super-pangenomic study including *B. fistulosa* would be of significant value in wax gourd breeding. Notably, Yang *et al.* (2023) identified seven accessions among the 146 accessions they analyzed that exhibited distinct gene PAV patterns from all other accessions, and excluded those seven accessions from part of their analysis as “abnormal samples”. This raises a concern that these “abnormal samples” may actually be *B. fistulosa* misidentified as *B. hispida*, which went unnoticed due to the absence of an outgroup in their analyses.

From an evolutionary genomics perspective, comparative genomics of the genus *Benincasa* with closely related genera would be of interest in understanding genome size dynamics not mediated by polyploidization. In several phylogenetic and phylogenomic studies, the genus *Benincasa* consistently formed a clade with the genera *Blastania*, *Dactyliandra*, and *Trochomeria* (Schaefer and Renner 2011, Schaefer *et al.* 2009, Zuntini *et al.* 2024, https://doi.org/10.1101/2023.10.27.564367). A comparative genomic study with these genera may elucidate the evolutionary history of the enlarged genome size of wax gourd.

### Bottle gourd

As in the cases of *Citrullus* spp. and wax gourd, the bottle gourd pangenome constructed to date is based solely on short reads and does not represent SVs other than gene PAVs (Table 1). A more intensive study of the bottle gourd pangenome using long-read sequencing technologies could reveal a comprehensive repertoire of SVs, including inversions, translocations, CNVs, more complex chromosomal rearrangements, and their effects on various traits.

There are also opportunities for comparative genomic or pangenomic studies involving wild relatives of bottle gourd. In addition to bottle gourd, the genus *Lagenaria* includes five other species (*L. abyssinica*, *L. breviflora*, *L. guineensis*, *L. rufa*, and *L. sphaerica*), all indigenous to tropical Africa. While these wild species may offer potential as breeding material for bottle gourd and as rootstock for other cucurbit crops, their genetic diversity is largely unexplored. Constructing a super-pangenome of the genus *Lagenaria* could reveal the untapped genetic diversity of these wild species, and provide further insight into how artificial selection has shaped bottle gourd, one of the earliest domesticated crops with diverse uses.

### Other cucurbits

As of June 2024, no pangenomic studies have been reported for cucurbits other than the five crops listed above. It is probably only a matter of time before such studies emerge, especially for major crops such as pumpkin/squash and bitter gourd. Phylogenetically, the five crops for which pangenomic studies have been reported all belong to the same tribe (Benincaseae) of the 15 tribes in the Cucurbitaceae family, and are relatively closely related to each other (Guo *et al.* 2020, Schaefer and Renner 2011, https://doi.org/10.1101/2023.10.27.564367). This arouses curiosity about the characteristics of the pangenomes of crops belonging to other tribes. In particular, *Cucurbita* spp. (Cucurbiteae) and chayote (Sicyoeae) are suggested to have undergone lineage-specific whole-genome duplication (Barrera-Redondo *et al.* 2019, Fu *et al.* 2021, Guo *et al.* 2020, Montero-Pau *et al.* 2018, Sun *et al.* 2017), which may result in distinct features in their pangenomes compared to other diploid cucurbits. However, analyzing these pangenomes would require special ingenuity due to the presence of paralogs in many of the genes.

## Prospects

The rise of pangenomics has shed light on the substantial diversity of SVs and their effects on various traits in a wide range of organisms, which are hardly accessible through conventional resequencing based on a single reference genome. Such comprehensive information on genetic variation promises to add another layer of accuracy and resolution to downstream applications such as linkage analysis, QTL mapping, GWAS, and genomic prediction.

Just as the development of sequencing technologies ushered in the pangenomic era in plants, we anticipate that continued technological innovation in the coming decades will further deepen plant pangenomics in two main directions. One direction is the extension of the pangenomic framework to higher taxonomic ranks such as genera and families, as is already happening in bacteria (Köstlbacher *et al.* 2021, Rajput *et al.* 2023). The other direction is the integration of pangenomes with other omics data such as transcriptome, epigenome, metabolome, and proteome. These advances will allow us to link more phenotypic variations than ever before to their underlying genetic variations, ultimately leading to the deciphering of the “blueprint of life” that we have desired since the dawn of the genomic era.

Different approaches have been used in pangenomic research on cucurbit crops, with results highlighting the inherent trade-off between scalability and resolution in short-read-based and long-read-based approaches (Table 1). Short-read-based approaches excel in scalability, allowing the integration of hundreds of samples into a study, but these approaches can only provide coarse information about individual SVs. In fact, all pangenomic studies on cucurbit crops that relied on short-read-based approaches focused only on genotyping gene PAVs based on mapped read coverage, and did not provide concrete information on the actual sequence variations underlying these gene PAVs. Conversely, long-read-based approaches are currently limited to relatively small-scale projects involving only a few dozen samples due to high sequencing costs and computational resource demands. However, these approaches enable the identification of any type of genetic variation at single-base resolution (Fig. 3), making them more promising than short-read-based approaches for translational applications in crop breeding, including marker-assisted selection, gene pyramiding, and targeted gene editing. Another important methodological note is that current pangenomic tools/pipelines sometimes fail to accurately genotype known SVs that have been confirmed to exist in plant genomes. This may be attributable to the fact that most tools/pipelines are primarily tested on non-plant genomes such as those of bacteria and humans (e.g., Garrison *et al.* 2018). Therefore, developing an analysis workflow that is specifically tailored to plant genomes and can cope with their large size, high repetitiveness, and frequent polyploidization would be an essential step in advancing the field of plant pangenomics.

It is noteworthy that many of the pangenomic studies of cucurbit crops consistently show that relatively primitive landraces or wild relatives possess genetic diversity absent from the improved or cultivated gene pool. Specifically, seven large inversions unique to wild accessions were identified in cucumber (Li *et al.* 2022). In melon, many genes were lost in parallel during the improvement of the two subspecies (Sun *et al.* 2022). In watermelon, more than 400 Mb of non-reference sequences were identified from wild relatives, which is comparable to the genome size of cultivated watermelon (Sun *et al.* 2023). In wax gourd, more than 300 genes predicted to be related to defense responses were lost during domestication and improvement (Yang *et al.* 2023). In bottle gourd, the most ancestral African population was found to have significantly more genes than other populations (Zhao *et al.* 2024). Similar trends have been reported in other crops, such as soybean (Li *et al.* 2014, Zhuang *et al.* 2022) and tomato (Gao *et al.* 2019). These findings reaffirm the potential role of crop wild relatives in re-expanding the genetic diversity of crop species, which has been narrowed by continuous selection over thousands of years.

Recent advances in plant genome editing technologies are enabling more precise, efficient, and flexible mutagenesis in a wider range of species. Beyond knock-out of a single gene through non-homologous end joining, various technologies have been developed, including base editors for precise point mutations (Molla *et al.* 2021), targeted editing of organelle genomes (Arimura and Nakazato 2023), transformation-free methods (Imai *et al.* 2020), and knock-in of kilobase-scale fragments using engineered CRISPR-Cas endonucleases (Schreiber *et al.* 2024). Although the social implementation of these technologies still requires careful consideration and discussion for appropriate regulations (Tachikawa and Matsuo 2024), these advances hold great promise for utilizing advantageous traits from wild species in crop breeding without the need for crossbreeding. In this context, constructing a pangenome that encompasses a broad range of crop wild relatives will be fundamental for understanding the genetic mechanisms underlying unique traits in these species. However, such efforts would be hampered by the limited availability of crop wild relatives at genetic resource centers. Therefore, in this pangenomic era, a wider range of wild relatives should be considered as potential genetic resources for crop breeding, regardless of their current economic value and cross-compatibility with crop species. These resources should be collected by interdisciplinary teams, including breeders, taxonomists, and ecologists, and made publicly available at genetic resource centers.

## Author Contribution Statement

GS prepared the table and figures and drafted the manuscript. KT and KK reviewed and refined the manuscript.

## Figures and Tables

**Table 1.** Summary of nine pangenomic studies in cucurbit crops published as of June 2024

Crop/Species	Number of accessions*^a^*	Sequencing technology*^b^*	Construction approach	Reference*^c^*
Cucumber	12 (11)	PacBio, Illumina	Graph-based pangenome	Li *et al.* (2022)*
Melon	297 (0)	Illumina	Map-to-pan	Sun *et al.* (2022)*
	25 (25)	ONT, Illumina	Comparative *de novo*	Oren *et al.* (2022)
	3 (2)	PacBio	Graph-based pangenome	Vaughn *et al.* (2022)
	9 (1)	PacBio, Illumina	Graph-based pangenome	Lyu *et al.* (2023)
*Citrullus* spp.	400 (0)	Illumina	Map-to-pan	Sun *et al.* (2023)*
	547 (201)	PacBio, Illumina	Map-to-pan	Wu *et al.* (2023)
Wax gourd	146 (0)	Illumina	Map-to-pan	Yang *et al.* (2023)*
Bottle gourd	197 (146)	Illumina	Map-to-pan	Zhao *et al.* (2024)*

*^a^* Numbers in parentheses indicate the number of accessions newly sequenced in the study. *^b^* PacBio, Pacific Biosciences; ONT, Oxford Nanopore Technologies. *^c^* Asterisks denote representative studies for each crop shown in Fig. 2.
